# Akt inhibition improves irinotecan treatment and prevents cell emergence by switching the senescence response to apoptosis

**DOI:** 10.18632/oncotarget.6126

**Published:** 2015-10-15

**Authors:** Alexandra Vétillard, Barbara Jonchère, Marie Moreau, Bertrand Toutain, Cécile Henry, Simon Fontanel, Anne-Charlotte Bernard, Mario Campone, Catherine Guette, Olivier Coqueret

**Affiliations:** ^1^ Paul Papin ICO Cancer Center, INSERM U892, CNRS 6299, Angers University, Angers, France

**Keywords:** chemotherapy, senescence, irinotecan, Akt, drug resistance

## Abstract

Activated in response to chemotherapy, senescence is a tumor suppressive mechanism that induces a permanent loss of proliferation. However, in response to treatment, it is not really known how cells can escape senescence and how irreversible or incomplete this pathway is. We have recently described that cells that escape senescence are more transformed than non-treated parental cells, they resist anoikis and rely on Mcl-1. In this study, we further characterize this emergence in response to irinotecan, a first line treatment used in colorectal cancer. Our results indicate that Akt was activated as a feedback pathway during the early step of senescence. The inhibition of the kinase prevented cell emergence and improved treatment efficacy, both *in vitro* and *in vivo*. This improvement was correlated with senescence inhibition, p21waf1 downregulation and a concomitant activation of apoptosis due to Noxa upregulation and Mcl-1 inactivation. The inactivation of Noxa prevented apoptosis and increased the number of emergent cells. Using either RNA interference or p21waf1-deficient cells, we further confirmed that an intact p53-p21-senescence pathway favored cell emergence and that its downregulation improved treatment efficacy through apoptosis induction. Therefore, although senescence is an efficient suppressive mechanism, it also generates more aggressive cells as a consequence of apoptosis inhibition. We therefore propose that senescence-inducing therapies should be used sequentially with drugs favoring cell death such as Akt inhibitors. This should reduce cell emergence and tumor relapse through a combined induction of senescence and apoptosis.

## INTRODUCTION

Activated in response to chemotherapy treatments, the p53-p21 and p16-Rb pathways induce apoptosis or senescence to prevent cancer cell proliferation. Apoptosis relies on the direct or indirect activation of the Bax/Bak pro-apoptotic proteins by BH3-only regulators such as Noxa, Bim or Puma and the consequent caspase induction and cell death. Conversely, senescent cells are viable but their replicative potential is lost as a result of cell cycle arrest and concomitant activation of the mTor pathway [1, 2]. This suppression relies on p21waf1 and p16INK4 activation and on the permanent inhibition of E2F-responsive genes through Rb activation and heterochromatin formation [3]. It has been shown that efficient tumor suppression relies on senescent cells being cleared by immune cells [4-6]. However, it should be noted that human naevi expressing the Raf oncogene remain senescent for years [7]. Thus, immune clearance might not be a common feature of this suppressive pathway.

Although they are arrested, senescent cells are not inactive since they produce soluble factors known as the senescence-associated secretory phenotype (SASP) [8-10]. The activity of the SASP is complex; depending on the experimental conditions, it has either a suppressive [10, 11] or an oncogenic function [12-14]. Thus, contrary to apoptosis, senescence can generate cells that can potentially alter the microenvironment, and maybe in some cases allow auto or paracrine tumor escape. As compared to apoptosis, these observations have led authors to question the efficacy of senescence as a complete tumor suppressive mechanism [15]. Illustrating this hypothesis, it has been recently reported that in mice models, p53-mediated senescence induced the failure of doxorubicin treatment [16]. This was related to the inhibition of mitotic catastrophy and apoptosis by the senescence-mediated arrest. Thus, the question of whether senescence and apoptosis elicit an equivalent level of tumor suppression is an important issue that needs to be clarified.

We have recently shown that cells can adapt to senescence and escape as a dividing population, either during oncogene-induced senescence [17] or following chemotherapy-induced senescence (CIS) [18-20]. Importantly, cells that resist CIS grow in low adhesion conditions, invade a matrigel matrix and form tumors *in vivo* [18]. We have proposed that more aggressive cells exit this suppressive pathway, either because senescence was not complete or because of a phenotypic switch that reconstitutes a dividing population. Interestingly, these cells show dependency on the Mcl-1 pro-survival protein. Its depletion increased treatment efficacy and prevented cell emergence, indicating that apoptosis effectively improves treatment efficacy in comparison to senescence.

In the current study, we pursued these experiments on the characterization of CIS escape, with the aim of finding combination therapies that would prevent cell emergence. Irinotecan is a well-known topoisomerase I inhibitor used as a first line treatment in colorectal cancer. Unfortunately cancer cells escape rapidly [21], requiring second line treatments and targeted therapies to increase the time to progression [22]. Among several resistance mechanisms, compensatory feedback pathways play an essential role in enabling cell escape in response to targeted therapies [23-27]. To our knowledge, this remains to be described in the context of irinotecan treatment and CIS escape. In this study, we describe that the Akt kinase is activated during CIS and that its inactivation significantly enhanced irinotecan efficacy and prevented cell emergence. It is significant to note that this was explained by the inactivation of senescence and the concomitant activation of apoptosis. Irinotecan normaly induces CIS through p21waf1 expression, but Akt inhibition downregulated this pathway, leading instead to the activation of the Noxa pro-apoptotic protein, followed by its binding to Mcl-1 and the consequent induction of apoptosis. Using p21waf1 −/− cells, we observed more generally that the presence of an intact senescence pathway favored cell emergence which was significantly reduced when apoptosis was induced.

Therefore, although chemotherapy killed off the vast majority of colorectal cancer cells, some subpopulations survived this treatment to proliferate as more aggressive cells. We propose that Akt targeting should be considered in the future to reduce senescence and improve the treatment of irinotecan-refractory colorectal cancers through enhanced apoptosis.

## RESULTS

### Sn38 triggers senescence and activates Akt

**Figure 1:**
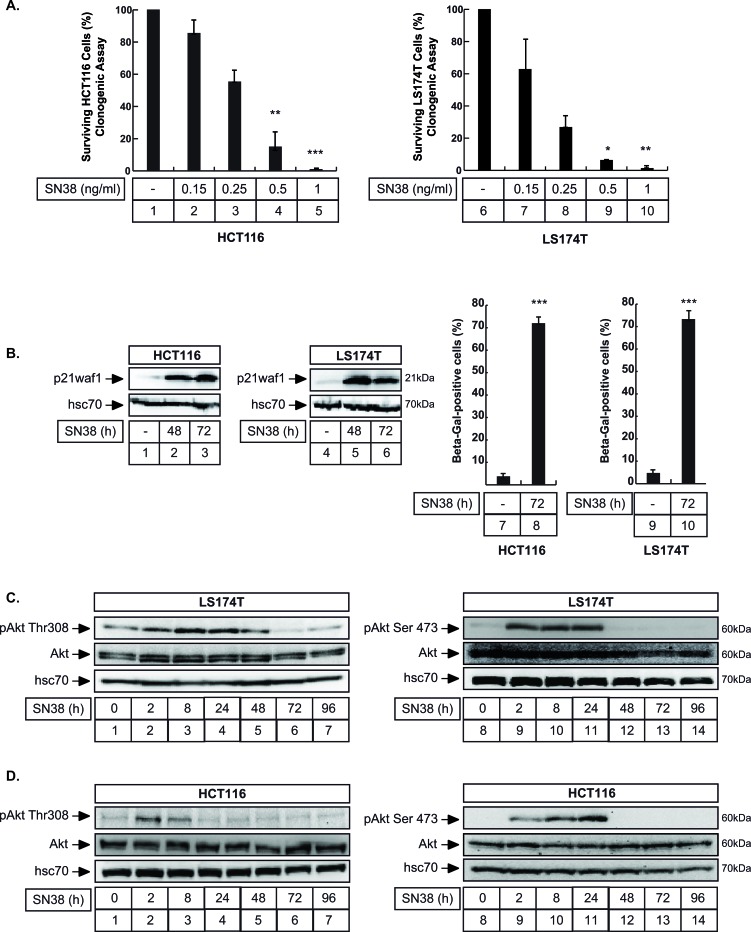
Akt is activated during Sn38-mediated senescence and cell cycle arrest **A.** HCT116 (left) and LS174T (right) cells have been treated with sn38 at the indicated concentrations and clonogenic assays were used to evaluate cell survival after 8-10 days of culture (*n* = 5 +/− sd, 1 ng/ml = 2.5 nM). **B.** LS174T and HCT116 cells have been treated with sn38 (5 ng/ml or 12.7 nM) for the indicated time, total cell extracts were then prepared and p21waf1 expression was evaluated by western blot (lanes 1-6, *n* = 4). Following sn38 treatment, the percentage of senescent cells was evaluated as the number of cells expressing SA-Δgal activity (*n* = 4 +/− sd). **C.**, **D.** LS174T (C) and HCT116 (D) cells have been treated with sn38 (5 ng/ml or 12.7 nM) for the indicated time, total cell extracts were then prepared and Akt activation was evaluated by western blot (*n* = 4).

Firstly, we confirmed our previous observations [18, 28], showing that sn38, the active metabolite of irinotecan, prevents the proliferation of colorectal cell lines and induces senescence and p21waf1 expression. Clonogenic assays performed on two different colorectal cell lines, LS174T and HCT116, confirmed that the number of colonies was reduced after treatment with sn38 (Figure 1A). Using western blot analysis, we observed an increase in p21waf1 expression after 48-72 hours of treatment (Figure 1B, lanes 1-6). Using β-galactosidase staining, a known marker of senescence, results indicated that approximately 70% of HCT116 and LS174T cells had entered senescence after 3 days (Figure 1B, lanes 7-10). Importantly, no signs of apoptosis were detected, analysing either caspase 3 activation or the presence of subG1 cells by flow cytometry (see below Figure 7).

Recent studies have shown that Akt signaling is used as a feedback survival pathway in response to targeted therapies [25, 27, 29]. Since this remains largely uncharacterized in response to irinotecan, we then determined whether this kinase was activated in response to sn38. Results presented Figure 1C and 1D indicate that Akt was phosphorylated on its threonine 308 and serine 473 residues in both HCT116 and LS174T cells. This active state was detected early, during the first 24 hours of treatment.

Altogether, we concluded from these results that the Akt kinase was activated in response to sn38 during the early stage of senescence induction.

### Akt inhibition enhances sn38 efficacy

**Figure 2:**
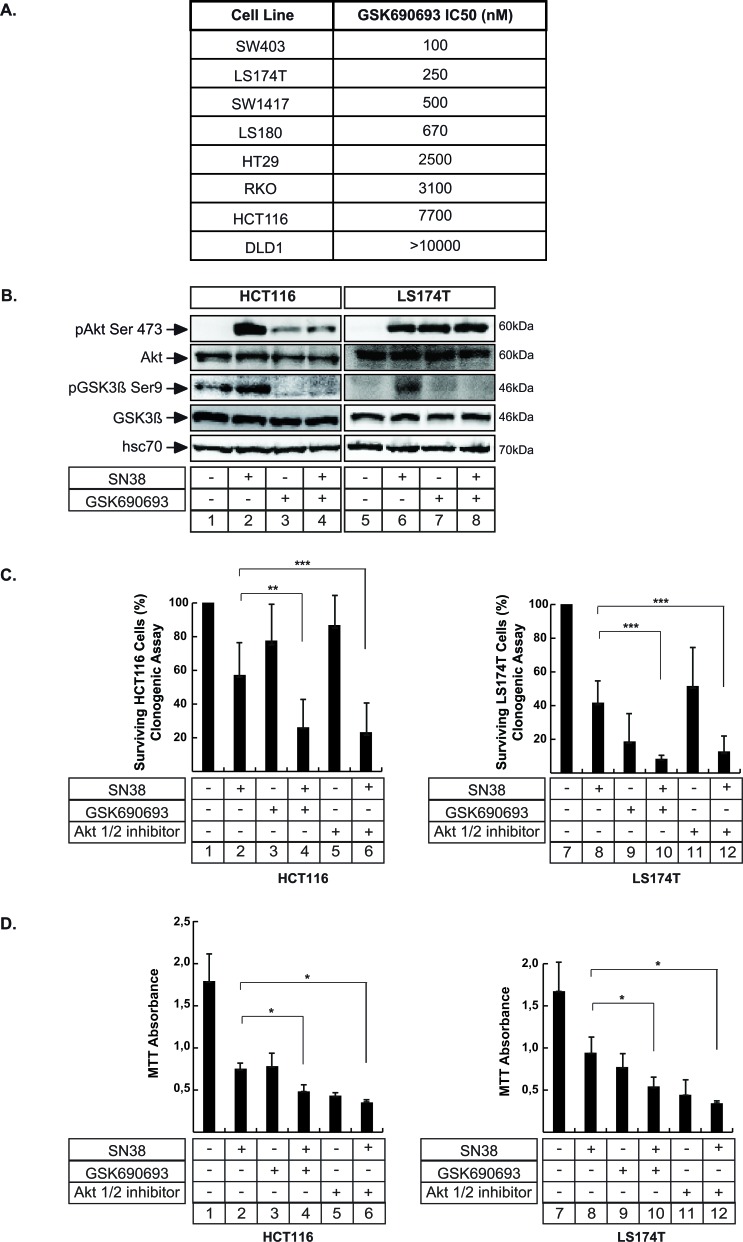
Akt inhibition enhances the effect of sn38 treatment **A.** The indicated cell lines were treated with different concentrations of GSK690693 and clonogenic assays were used to evaluate cell survival after 8-10 days of culture. IC50 values were calculated by counting the number of surviving colonies. **B.** LS174T and HCT116 cells have been treated with sn38 (5 ng/ml or 12.7 nM), GSK690693 (20μM) or both drug for 8hr. Akt and GSK3Δ expression and phosphorylation have been evaluated by western blot (*n* = 3). **C.** Following treatment with sn38 (0.25 ng/ml or 0.63 nM), GSK690693 (1μM for LS174T cells and 7μM for HCT116 cells), Akti 1/2 inhibitor (4μM), clonogenic assays were used to evaluated long term cell death after 7-10 days of culture (*n* = 8 +/− sd). **D.** LS174T and HCT116 cells have been treated with sn38 (5 ng/ml or 12.7 nM) in the presence or absence of GSK690693 (20 μM) or Akti 1/2 (10 μM) as indicated for 72hr. MTT assays were then used to evaluate short time effects on cell viability (*n* = 4 +/− sd).

Since Akt was activated in response to sn38, we used selective inhibitors of the kinase to determine whether its inactivation could improve the efficacy of the topoisomerase inhibitor. To this end we first used GSK690693, which has been described recently as a specific ATP-competitive inhibitor of the kinase [30]. Using a panel of representative colorectal cell lines and clonogenic assays, we observed that the sensitivity varied, depending on the cell type (Figure 2A). For example, LS174T cells were sensitive to GSK690693 with a low IC50 of 250 nM whereas HCT116 cells were more resistant with an IC50 closer to 7700 nM (an equivalent high IC50 was obtained in ref [30] using short time proliferation assays. See the material and methods section for a description of the doses and survival assays used in this study). Using these two representative cell lines, we then determined whether GSK690693 effectively blocked Akt signaling following sn38 treatment. To this end, cells were treated with sn38, GSK690693 or both and GSK3β phosphorylation was analyzed since this protein is one of the main targets of Akt. The results presented in Figure 2B show a decreased GSK3β phosphorylation, confirming Akt inhibition (note the increase of Akt phosphorylation detected following GSK690693 treatment, lanes 3 and 7-8. This has been previously reported in [30]). We then determined whether this inhibitor could improve the efficacy of sn38. Firstly, we used clonogenic assays to evaluate long term cell death/arrest, by treating the cells with sn38 with or without GSK690693 added at the same time. As shown in Figure 2C (compare lanes 2 and 4; 8 and 10), the treatment with both drugs significantly decreased the percentage of clones in HCT116 and LS174T cells. This observation was confirmed using MTT assays (Figure 2D, compare lanes 2 and 4; 8 and 10). In addition, the same effect was also observed with Akti 1/2, a non-ATP-competitive and PH-domain dependent Akt inhibitor (Figure 2C and 2D, compare lanes 2 and 6, 8 and 12). Note that MTT assays give information on cell division but also on cell viability and growth. Given the importance of Akt/mTor on these different pathways, further experiments are necessary to interpret completly these MTT results.

Altogether, these results show that the pharmacological inhibition of Akt potentiates the effect of sn38.

### Akt inhibition prevents cell emergence and treatment escape

**Figure 3:**
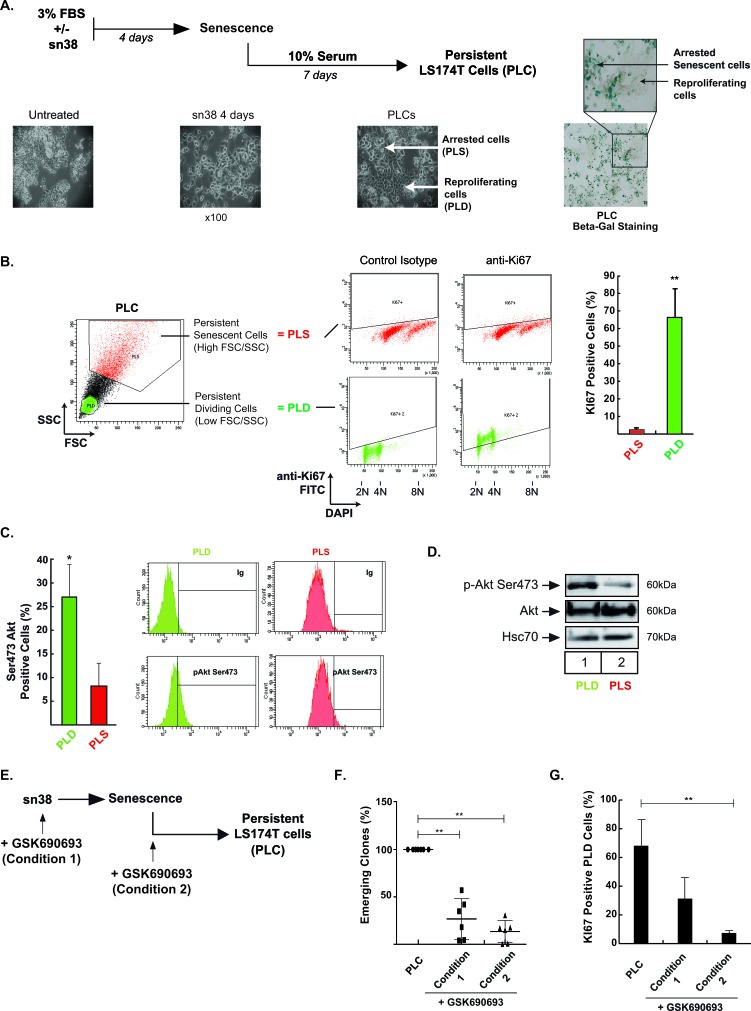
Akt inhibition prevents cell emergence and treatment escape **A.** Experimental procedure to generate persistent LS174T cells named PLC in this study: following sn38 treatment (5 ng/ml or 12.7 nM, 96 hrs), cells have been stimulated with 10% FBS for 7 days to reinduce cell growth. Representative images showing PLC heterogeneity are shown on the right, illustrating the presence of dividing (PLD) clones within senescent cells (PLS) stained with Δ-galactosidase. **B.** Following DAPI staining and flow cytometry analysis, PLC cells were gated according to low (PLD) or high (PLS) FSC/SSC values. Ki67 expression was evaluated in each subpopulation. Cell percentages are presented on the right side (*n* = 5 +/− sd). **C.** pAkt Ser473 phosphorylation was analyzed by flow cytometry in PLC. Cells were gated according to low and high FSC/SSC values and the corresponding pAkt Ser473 expression was evaluated in PLD and PLS (*n* = 4 +/− sd). Representative images are shown on the right. **D.** PLD and PLS cells have been cell sorted by flow cytometry according to low or high FSC/SSC values, as described in [18]. Akt phosphorylation has been evaluated by western blot in the indicated cells (*n* = 3). **E.**, **F.** PLC were generated in the presence or absence of GSK690693 added at different times during emergence, either at the same time as sn38 (condition 1) or at the time of release in 10% serum (condition 2). Emerging clones were counted using crystal violet staining and compared to PLC generated in the absence of Akt inhibition (*n* = 6 +/− sd). **G.** In the same conditions, PLD and PLS cells were gated according to low and high FSC/SSC values and the amount of proliferating cells was evaluated in PLD cells using Ki67 expression (*n* = 6 +/− sd).

We have recently described that subpopulations of colorectal cells can adapt to senescence and chemotherapy and resume proliferation [18-20, 31]. Escape to sn38 leads to the emergence of more transformed cells that we have named PLC (Persistent LS174T Cells, see Figure 3A). These emergent cells are more aggressive than parental cells, they induce tumor formation in mice, grow in low adhesion conditions and resist anoikis [18]. The PLC population is heterogeneous and composed of around 60-70% senescent cells (named PLS) and 30-40% of proliferating cells (named PLD). Representative images presented in Figure 3A describe both this emergence and PLC heterogeneity. Illustrative Δ-galactosidase staining of the two sub-populations is shown on the right of Figure 3A (note the presence of PLD proliferating subclones in the middle of the arrested PLS cells). Using flow cytometry analysis and cell sorting, we have described that we can identify the PLD sub-population within the PLC according to a low size and granularity profile and a high KI-67 staining [18]. Results presented Figure 3B confirmed that cells with a low FSC/SSC profile divided efficiently as evidenced by a high KI-67 staining (FSC: Forward-scattered light, proportional to cell-surface area or size, SSC: Side-scattered light, proportional to cell granularity). By contrast a high FSC/SSC profile was associated with the absence of KI-67 expression. We have previously shown that this subpopulation was mainly composed of PLS senescent cells [18].

**Figure 4:**
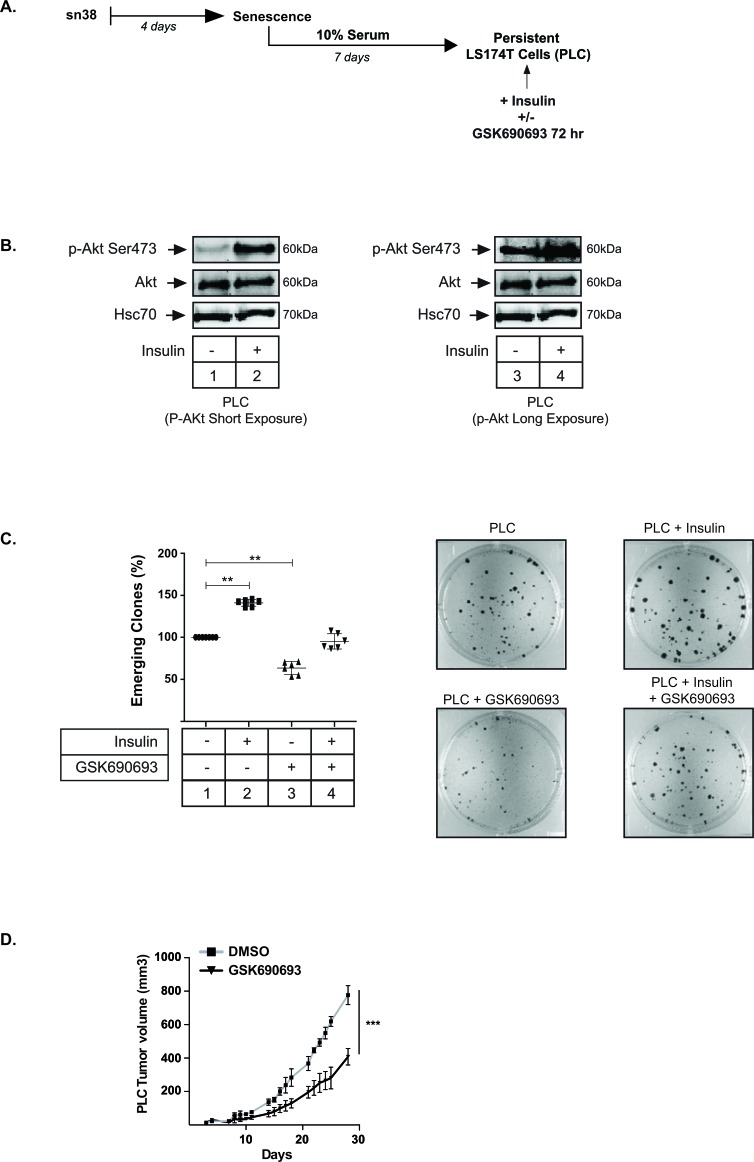
Akt activation in PLC increases the number of PLD **A.** Persistent LS174T cells (PLC) were generated and then were treated with Insulin (500 nM) and/or GSK690693 (20 μM) for 72 hrs. **B.** PLC were generated, treated with Insulin and Akt activation was evaluated by western blot (*n* = 4). **C.** PLC were generated, treated with Insulin and/or GSK690693 and the number of clones were counted using crystal violet staining and compared to PLC without treatment, lane 1 (*n* = 6 +/−sd). Representative images are shown on the right. **D.** PLC were generated and injected subcutaneously in immunocompromised mice. After 4 days, mice were treated or not with GSK690693 (10mg/kg), 5 days per week. The tumor volume was monitored over 4 weeks.

This model of sn38 escape was used to determine whether Akt was implicated in the emergence of PLD cells. Using intracellular flow cytometry staining, we observed that around 25% of dividing PLD express the Ser473 phosphorylated form of Akt. By contrast the kinase was almost not activated in the non-dividing PLS subpopulation (Figure 3C). This difference was confirmed by western blot, using cell sorting and extracts obtained from the PLD or PLS subpopulations. The active form of Akt was mainly detected in the PLD as compared to the senescent PLS cells (Figure 3D). This activation of Akt in the PLD subpopulation suggested to us that the kinase could play a role in the re-activation of cell division during emergence. To test this hypothesis, we blocked Akt activation at various points in time using GSK690693. The drug was added either at the same time as sn38 (condition 1, Figure 3E), or at the time of release in 10% serum (condition 2, Figure 3E). Results presented in Figure 3F indicate that Akt inhibition significantly limited the emergence of clones detected by crystal violet staining after 7-10 days. Using KI67 staining, we found that this led to a significant reduction of PLD KI67-positive cells (Figure 3G). We then determined if Akt was able to modify the proportion of PLS and PLD cells at the end of emergence. To avoid potential artefacts due to DNA transfection following cell sorting, we generated PLC and then stimulated the cells with insulin, a well known activator of Akt (see protocol Figure 4A). Western blot experiments confirmed that the kinase was phosphorylated in this condition (Figure 4B, compare lanes 1 and 2, lanes 3-4 correspond to a higher exposition to show the activation of the kinase in PLC, lane 3). Interestingly, insulin induced a significant increase in the number of dividing clones after 72 hr. This effect was blocked by GSK690693, confirming the role of the kinase in the generation of emergent cells (Figure 4C, compare lanes 2 and 4). As expected, GSK690693 used alone reduced the number of PLD (Figure 4C, compare lanes 1 and 3). To extend these observations, we then asked if Akt inhibition prevents the growth of PLC *in vivo*. To this end, PLC were generated and then injected subcutaneously into immunocompromised mice. Starting at day 4, mice were treated or not with GSK690693 (10mg/kg, 5 days per week for 4 weeks). The results presented in Figure 4D indicate that the Akt inhibitor was able to significantly reduce tumor formation by PLC *in vivo*.

Overall, we concluded from these results that the pharmacological inhibition of Akt reduced cell emergence and senescence escape in response to sn38.

### Akt inhibition prevents p21waf1 and senescence induction

**Figure 5:**
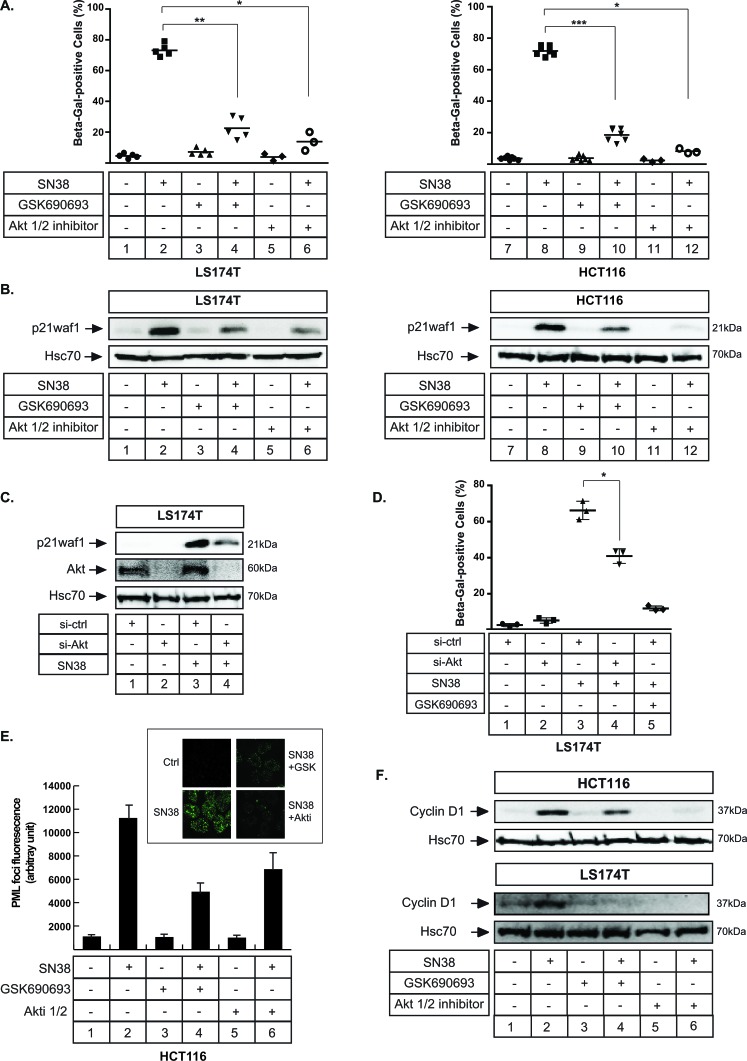
Akt inhibition prevents p21waf1 expression and senescence induction **A.** LS174T and HCT116 cells have been treated with sn38 (5 ng/ml or 12.7 nM) in the presence or absence of GSK690693 (20 μM) and Akti 1/2 (10 μM) as indicated for 72 hrs and the percentage of Δ-galactosidase-positive cells was evaluated (*n* = 4 +/−sd). **B.** LS174T and HCT116 cells have been treated as above, total cell extracts were prepared and p21waf1 expression was evaluated by western blot (*n* = 5). **C.** Akt expression was down-regulated by RNA interference, the next day LS174T cells were treated with sn38 during 72 hours, total cell extracts were then prepared and p21waf1 and Akt expression were evaluated (*n* = 3). **D.** Akt expression was down-regulated by RNA interference, the next day LS174T cells were treated with sn38 during 72 hours and the percentage of Δ-galactosidase-positive cells was evaluated (*n* = 4 +/− sd). **E.** HCT116 cells were treated with sn38 in the presence or absence of GSK690693 and Akti 1/2 for 72 hrs. PML staining was then evaluated by immunofluorescence. The average of fluorescence intensity per cell has been quantified (*n* = 3). **F.** LS174T (bottom panel) and HCT116 (top panel) cells have been treated as above, total cell extracts were prepared and cyclin D1 expression was evaluated by western blot (*n* = 3).

Since Akt inhibition prevented cell emergence, we then asked whether this was related to the regulation of senescence. We firstly used β-galactosidase staining to test this hypothesis and we observed that sn38-mediated senescence was significantly reduced when Akt was inactivated. This result was obtained with both Akt inhibitors, in LS174T and HCT116 cells (Figure 5A compare lanes 2 and 4, 6 and lanes 8 and 10,12). In addition, western blot analysis showed that p21waf1 induction was decreased when Akt was inhibited (Figure 5B, compare lanes 2 and 4, 6 and lanes 8 and 10, 12). This was expected since the kinase is known to regulate the stability and localisation of the cell cycle inhibitor [32-34]. Cell fractionation experiments indicated that p21waf1 downregulation occured in both cell compartments without any cytoplasmic relocalization (data not shown). This inhibition was confirmed using RNA interference to downregulate Akt. Following sn38 treatment, p21waf1 was induced as expected in the presence of a control siRNA. Its expression was reduced when cells were transfected with a siRNA directed against the kinase (Figure 5C, compare lanes 3 and 4). Akt was downregulated as expected. Using β-galactosidase staining, we also confirmed using RNA interference that Akt inhibition reduced sn38-mediated senescence as compared to cells transfected with a control siRNA (Figure 5D, compare lanes 3 and 4, the same effect was obtained in HCT116 cells (data not shown)). The inhibition was less efficient as compared to the one obtained with GSK690693 (Figure 5D, lane 5), probably as a consequence of an incomplete downregulation of the kinase. We then determined the effect of Akt inhibition on PML (promyelocytic leukemia) bodies, nuclear structures that are associated with senescence and genotoxic stress [35]. As expected, PML bodies were detected after 72 hr of sn38 treatment. Interestingly, Akt inhibition reduced their number and this effect was observed with the two different inhibitors, GSK690693 and Akti 1/2 (Figure 5E, compare lanes 2 and 4, 2 and 6). To further confirm the inhibition of the senescence pathway, we then analyzed the expression of cyclin D1. This protein has recently been described as an additional marker of senescence since its level increased in response to several senescence-inducing drugs [36-38]. We were able to confirm this observation in different cell lines (data not shown and the upregulation of cyclin D1 during sn38-mediated senescence is presented Figure 5F, lanes 1-2). Interestingly, like p21waf1, this protein was also downregulated when Akt was inactivated. This effect was observed in LS174T cells and with the two different inhibitors (Figure 5F, compare lanes 2 and 4, 6). The inhibition was less obvious in HCT116 cells.

Thus, we concluded from these observations that the inhibition of Akt prevents senescence induction in response to sn38.

### The presence of p21waf1 is necessary for cell emergence in response to sn38

**Figure 6:**
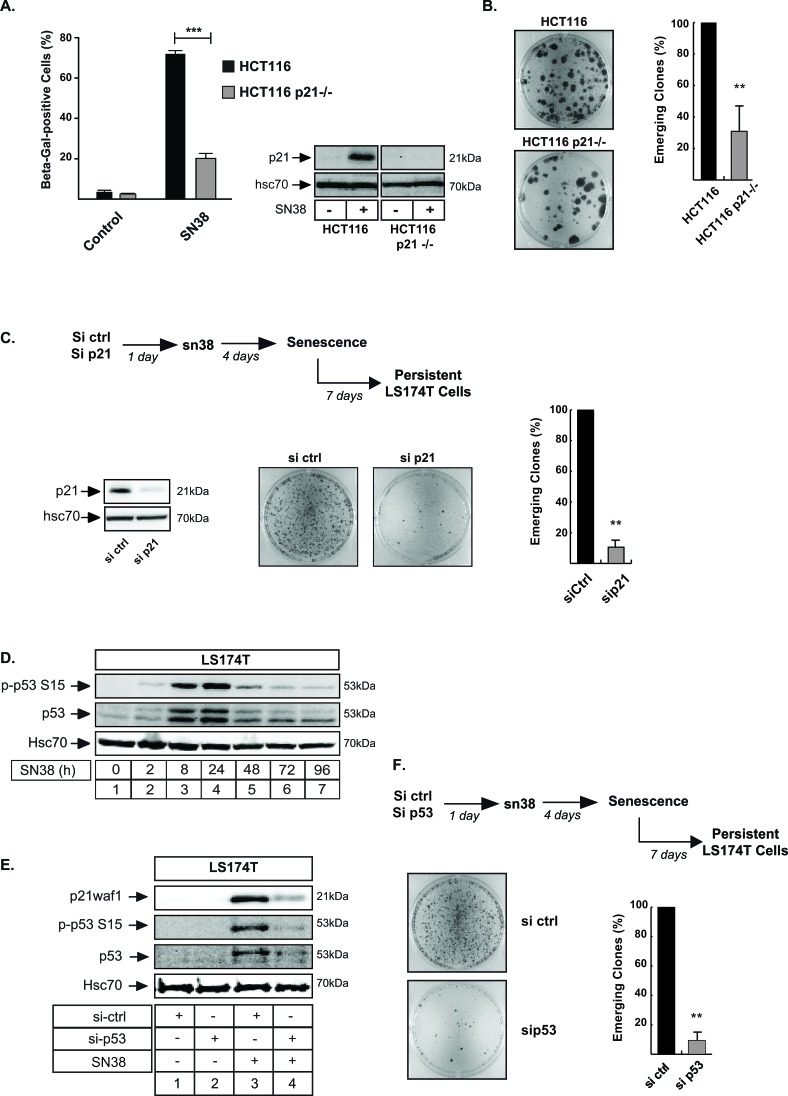
The presence of p21waf1 is necessary for cell emergence **A.** HCT116 and HCT116 p21−/− cells have been treated with sn38 (5 ng/ml or 12.7 nM) and the percentage of Δ-Galactosidase positive cells was evaluated after 72 hrs (*n* = 4 +/− sd). **B.** Emergence was evaluated in HCT116 and HCT116 p21−/− cells following sn38 treatment. Note that in comparison with LS174T cells, emergence occurred after more than 14 days in this cell line. Clones were then counted using crystal violet staining (*n* = 5 +/− sd). **C.** p21waf1 expression was downregulated by RNA interference, in LS174T cells. PLC were then generated as above and emergence was evaluated by crystal violet staining (*n* = 5 +/− sd). **D.** LS174T cells were treated with sn38 (5 ng/ml or 12.7 nM) for the indicated time, total cell extracts were then prepared and p53 activation was evaluated by western blot with the indicated antibodies (*n* = 5). **E.** p53 expression was downregulated by RNA interference, the next day LS174T cells were treated with sn38 for 48 hours, total cell extracts were then prepared and p21waf1 and p53 expressions were evaluated (*n* = 3). **F.** p53 expression was down-regulated by RNA interference, PLC were generated and cell emergence was evaluated as compared to cells transfected with a control siRNA. Representative images of crystal violet staining are shown (*n* = 5 +/− sd).

We were surprised that a better sn38 response was observed in parallel to p21waf1 and senescence inhibition. As the main mediator of sn38-mediated arrest, we expected this pathway to be maintained, leading to a complete loss of replicative potential [1]. To explain this observation, we analyzed cell emergence in the absence of the cell cycle inhibitor. Using first HCT116 p21−/− cells, we confirmed that sn38-mediated senescence was significantly reduced as compared to parental cells (Figure 6A). Interestingly, p21waf1 inactivation significantly reduced the number of persistent clones in response to sn38 (Figure 6B). These results were confirmed by a second approach in LS174T cells, using RNA interference to downregulate p21waf1. Again, and as compared to cells transfected with a control siRNA, p21waf1 inactivation significantly reduced the number of PLC clones (Figure 6C). Western blot analysis confirmed the downregulation of the cell cycle inhibitor (Figure 6C, left). Since p21waf1 is activated by p53 in response to genotoxic treatment [39, 40], we also analyzed the role of this tumor suppressor during cell emergence. As expected, p53 was upregulated and phosphorylated on its Ser15 residue following sn38 treatment (Figure 6D). Results also confirmed that its inactivation by RNA interference prevented p21waf1 activation (Figure 6E, compare lanes 4 and 3). Significantly, almost no cells were able to escape sn38 treatment when p53 was downregulated. Representative images of crystal violet staining and the quantification of emergence are shown Figure 6F.

Altogether, we concluded from these results that the inactivation of the p21waf1-p53 senescence pathway reduced cell emergence and improved treatment efficacy.

### Apoptotic cell death reduces cell emergence following p21waf1 downregulation

**Figure 7:**
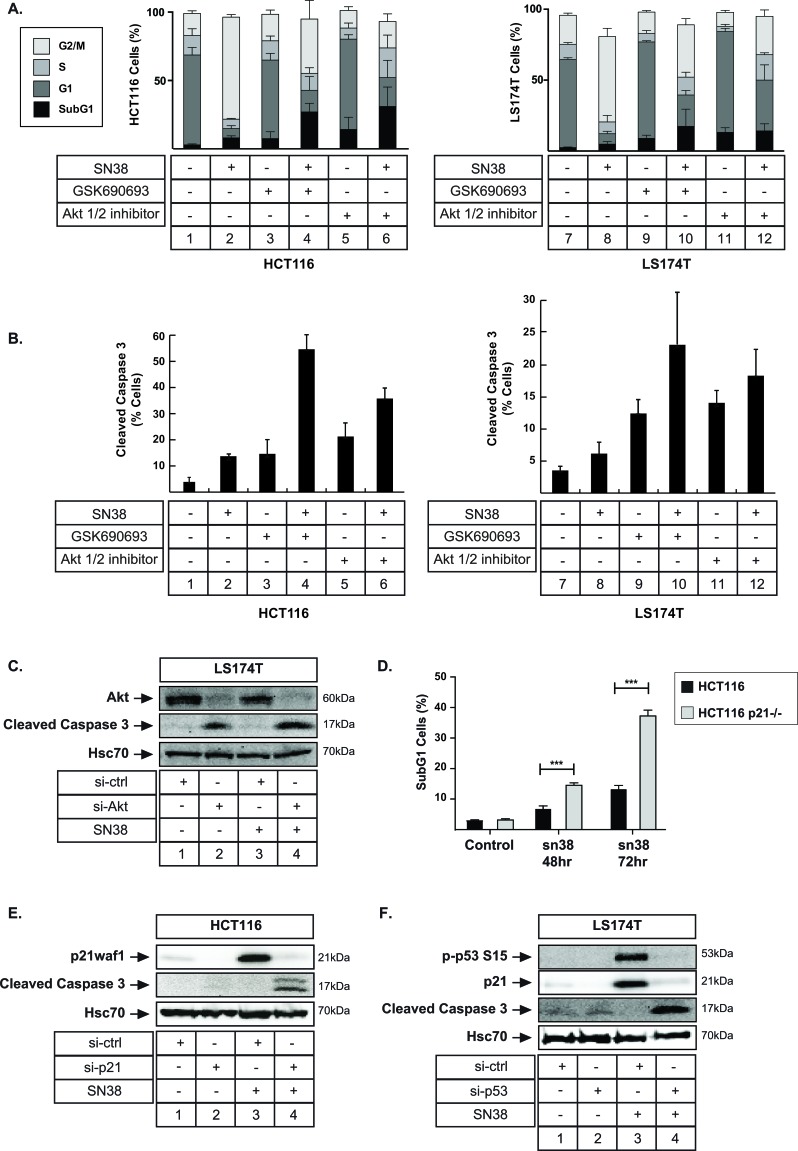
Apoptotic cell death is induced following senescence inhibition **A.** HCT116 and LS174T cells were treated as above with sn38 (5 ng/ml or 12.7 nM), GSK690693 (20 μM) or Akti ½ (10 μM) for 72h. Flow cytometry experiments were then performed to quantify the percentage of cells in each phase of cell cycle (*n* = 4 +/− sd). **B.** Cells were treated as above and apoptosis was evaluated by FACS analysis and the detection of the active form of caspase 3 (*n* = 3 +/− sd). **C.** Akt was downregulated by RNA interference, the next day LS174T cells were treated with sn38 (5 ng/ml or 12.7 nM), total cell extracts were then prepared and the expression of the cleaved caspase-3 was evaluated by western blot (*n* = 3). **D.** HCT116 and HCT116 p21−/− cells were treated with sn38 (5 ng/ml or 12.7 nM) for the indicated time and the percentage of cells presenting a subG1 content was evaluated by FACS analysis (*n* = 5 +/− sd). **E.** p21waf1 was downregulated by RNA interference, the next day HCT116 cells were treated with sn38 (5 ng/ml or 12.7 nM) for 48 hours, total cell extracts were then prepared and the expression of the cleaved caspase-3 was evaluated by western blot (*n* = 3). **F.** p53 expression was downregulated by RNA interference, LS174T cells were treated as above and the expression of the cleaved caspase-3 was evaluated (*n* = 3).

As stated in the introduction, elegant results using mice-models have reported that p53-mediated arrest and senescence induce chemotherapy failure [16]. It was proposed that an efficient cell cycle arrest prevents mitotic catastrophy and consequently reduces cell death and treatment efficacy. It is also known that p21-deficient tumors are more sensitive to radiotherapy *in vivo* [41, 42]. In light of these studies, we therefore sought to determine whether Akt inhibition and p21waf1 downregulation reduced cell emergence through apoptosis. As we previously described [18, 43], flow cytometry analysis showed that sn38 induced a G2/M arrest. Interestingly, Akt inhibition reduced this percentage of G2/M cells and increased the proportion of subG1 cells. This was observed both in HCT116 and LS174T cells and with the two inhibitors (Figure 7A, compare lane 2 with 4, 6 and 8 with 10, 12, note that more subG1 cells were detected in HCT116 cells). This induction of apoptotic cell death was further validated by the detection of the active form of caspase 3 by flow cytometry. Although less than 15% of cells presented an active form of caspase 3 following sn38 treatment, this percentage increased in the presence of GSK690693 or Akti 1/2 (Figure 7B, compare lanes 2 with 4, 6 and 8 with 10, 12). The same effect was observed when Akt was inactivated by RNA interference in LS174T (Figure 7C, lane 4) and HCT116 cells (data not shown).

We then asked if this apoptotic cell death was related to the downregulation of p21waf1. As described Figure 7B, when HCT116 cells were treated with sn38, apoptosis was detected in less than 15% cells. By contrast, when these experiments were performed in HCT116 p21−/− cells, a significant increase in the number of subG1 cells was observed (Figure 7D). The activation of caspase 3 was also detected by western blot when p21waf1 was inactivated by RNA interference in HCT116 cells (Figure 7E, compare lanes 4 and 2). In addition, the downregulation of p53 also led to caspase 3 activation in response to sn38 (Figure 7F, compare lanes 4 and 2).

In light of these results, we concluded that Akt inhibition improved sn38 efficacy and prevented cell emergence through the induction of apoptosis.

### Noxa inhibits Mcl-1 to prevent cell emergence

**Figure 8:**
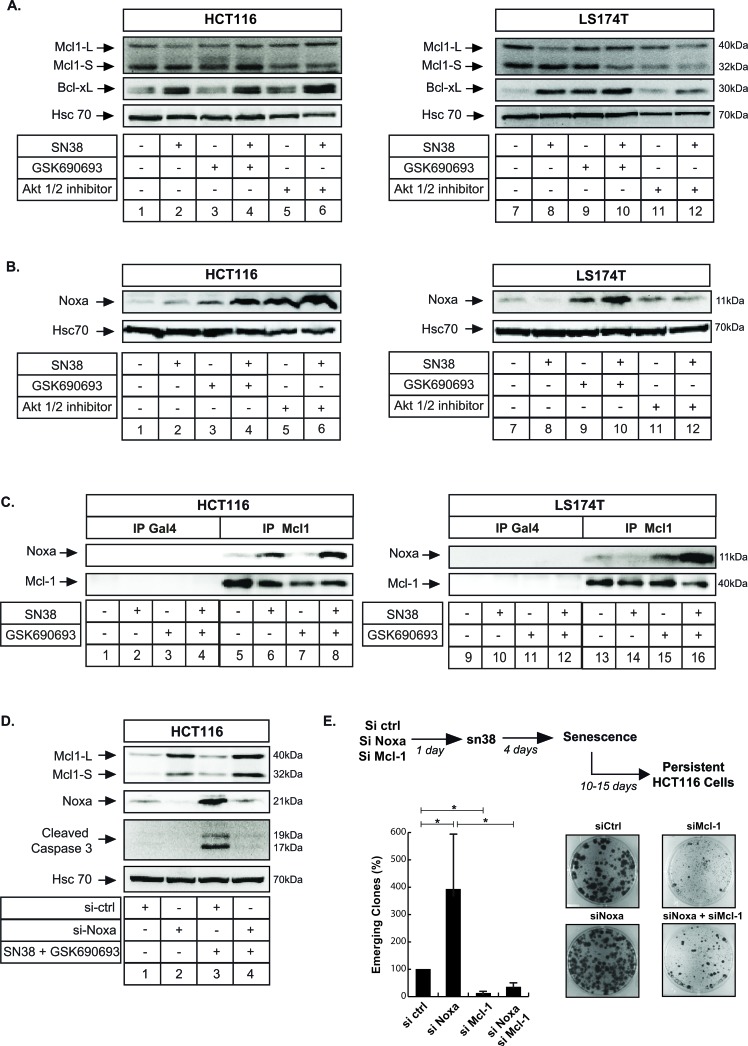
Noxa interacts with Mcl-1 to induce apoptosis and prevent cell emergence in response to Akt inhibition **A.**, **B.** Cells have been treated with sn38, GSK690693 or Akti 1/2 inhibitor as above for 72 hr, total cell extracts were then prepared and Mcl-1 (A), Bcl-xL (A) and Noxa (B) expressions were evaluated by western blot (*n* = 3). **C.** Cells have been treated with sn38 and GSK690693 as above for 72 hr, total cell extracts were then prepared and the interaction between Mcl-1 and Noxa was evaluated by co-immunoprecipitation. An antibody directed against the Gal4 protein was used as a control (*n* = 4). **D.** Noxa was downregulated by RNA interference, the next day HCT116 cells were treated with sn38 and GSK690693 for 48 hours, total cell extracts were prepared and the expression of the active form of caspase 3 was evaluated (*n* = 3). **E.** Noxa or Mcl-1 expressions were downregulated by RNA interference, persistent HCT116 cells were then generated and cell emergence was evaluated by crystal violet staining after 10-15 days (*n* = 5 +/−sd).

We and others have recently reported that Mcl-1 favors senescence escape and cell emergence [18, 20, 31, 44]. To determine whether Akt inhibition prevents sn38 escape through the downregulation of this survival protein, we first analyzed its expression by western blot. GSK690693 or Akti 1/2 did not affect Mcl-1 level in HCT116 cells and we did not obtained any reproducible effect in LS174T cells (Figure 8A). Although previous experiments have reported that Mcl-1 levels are regulated by the Akt-GSK3β pathway [45], this was not the case in our experimental conditions. In addition, no effect was observed on Bcl-xL which we and others have previously shown to be upregulated in response to sn38 [18, 46] (Figure 8A).

Besides its downregulation, Mcl-1 is also inactivated following its titration by Noxa [47]. In response to sn38, we found that this pro-apoptotic protein was weakly induced in HCT116 but not in LS174T cells (Figure 8B, compare lanes 1 and 2; 7 and 8, see also ref [48]). Interestingly, Noxa was significantly upregulated in the two cell lines following Akt inhibition (Figure 8B, compare lanes 2 and 4, 6; 8 and 10, 12). Using co-immunoprecipitation analysis, we observed that Noxa weakly interacted with Mcl-1 in HCT116 cells following sn38 treatment. This interaction was further enhanced when Akt was inhibited (Figure 8C, compare lanes 8 and 6). In LS174T cells, the combined use of sn38 and GSK690693 also induced a significant binding of Noxa to Mcl-1 (Figure 8C, compare lanes 14 and 16).

If Noxa mediates apoptosis following Akt inhibition, we reasoned that its inactivation should prevent cell death and favor cell emergence. We first tested this hypothesis by downregulating Noxa and analyzing caspase 3 activation in the presence of sn38 and GSK690693. As expected, the combined treatment induced caspase 3 activation when cells were transfected with a control siRNA, (Figure 8D, compare lanes 3 and 1). Interestingly, Noxa downregulation prevented this activation (Figure 8D, compare lanes 4 and 3).

We then determined whether Noxa downregulation and cell death inhibition led to increased cell emergence. To test this hypothesis, this protein was inactivated by RNA interference, cells were then treated with sn38 and emergence was evaluated after 15 days (See Figure 8E top). We did this experiment on HCT116 since these cells express Noxa in response to sn38 which was not the case for LS174T cells (Figure 8B, lane 2 and 8). The results presented in Figure 8E indicate that Noxa inactivation effectively enhanced the number of emergent cells, in comparison with cells transfected with a control siRNA. Interestingly, and as previously reported [49, 50], we noticed that Noxa inhibition led to an increase in Mcl-1 expression (Figure 8D, lanes 2 and 4). This suggested that this survival protein might explain cell escape in this condition as we previously reported [18, 20]. To test this hypothesis and to further determine if the effect of Noxa are indeed mediated by Mcl-1 inhibition, we downregulated this pro-survival protein by RNA interference. As expected, we observed that Mcl-1 inhibition reduced the number of emergent cells [18]. Interestingly, results also indicated that the increased emergence observed in the absence of Noxa was completely inhibited when Mcl-1 was downregulated (Figure 8E).

Altogether, we concluded from these results that Akt inhibition prevents sn38 persistence through Noxa activation, Mcl-1 binding and apoptosis induction.

### Apoptosis can still be induced when senescence is established

**Figure 9:**
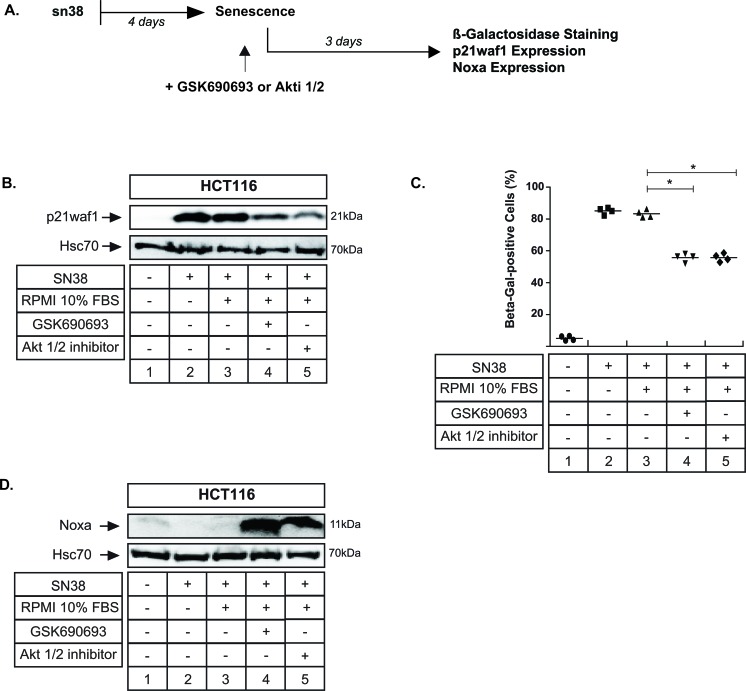
Apoptosis can still be induced when senescence is already established **A.** LS174T cells have been treated with sn38 (5 ng/mL or 12.7 nM) for 96 hrs (lane 2), serum was added (lane 3) in the absence or presence of GSK690693 (20μM, lane 3) or Akti 1/2 (10μM, lane 4) for 3 days in 10% serum. **B.** In these experimental conditions, total cell extracts were prepared, and p21waf1 expression was then evaluated by western blot (*n* = 4). **C.** In the same condition, the percentage of Δ-galactosidase-positive cells was evaluated (*n* = 4, +/− sd). **D.** In these experimental conditions, total cell extracts were prepared, and p21waf1 expression was then evaluated by western blot (*n* = 4).

We then determined if we could reduce senescence when this suppressive mechanism was already activated. To this end, cells were pretreated with sn38 for 4 days and Akt was inhibited for three days after this initial treatment (see the experimental approach Figure 9A, RPMI 10% FBS was added as a control). Senescence was detected through p21waf1 expression and β-Galactosidase staining (Figure 9B, compare lanes 2 and 1; Figure 9C, compare lanes 2 and 1). Interestingly, when Akt was inhibited in this senescent population, we still observed that p21waf1 expression could be downregulated (Figure 9B, compare lanes 4-5 with 3). In addition, we also noticed a significant decrease in β-Galactosidase staining (Figure 9C, compare lanes 4-5 with 3). Western blot experiments indicated that Noxa was also induced when the kinase was inhibited in senescent cells (Figure 9D, compare lanes 4 and 5 with 2 and 3). These effects were observed with the two different Akt inhibitors, GSK690693 and Akti 1/2.

In light of these results, we concluded that Akt inhibition can still reduce senescence and upregulate Noxa when this suppressive pathway is already induced. Since the definition of senescence implies that it is a definitive mechanism, this might suggest that the Akt inhibitors target a subpopulation of cells where senescence is not complete.

## DISCUSSION

Understanding intrinsic or adaptive resistance pathways in response to chemotherapy is one of the main challenges of cancer treatment. Progression can be explained by tumor heterogeneity and by the presence of pre-existent resistant cells. Intrinsic drug resistance, abnormal drug transport and detoxification enzymes or enhanced DNA repair can all favor the selection of specific subclones [23]. In addition, cancer cells can also adapt to chemotherapy during the course of the treatment. For instance, the epithelial-mesenchymal transition (EMT) and the consequent generation of cells with cancer-initiating cells features represent an important source of therapeutic failure [51-53] [54]. These dedifferentiation pathways are used by cancer cells in stressful conditions to finally reconstitute dividing populations. Therefore, a complete eradication will certainly rely on combination therapies that can kill at the same time the bulk of the sensitive tumor and the persistent clones.

To understand these adaptive mechanisms, we have recently developed two models of senescence escape, either in response to the Ras oncogene [17] or during chemotherapy-induced senescence (CIS) [18-20]. In both cases, we have observed that a subpopulation of cells can escape senescence and emerge as a more aggressive, dividing population. It is striking that in these two cases, the persistent cells depend on Bcl-xL and/or Mcl-1, two survival proteins of the Bcl-2 family. This suggests that during the initial senescence induction or during the latter stages of tumor escape, apoptosis is a fail-safe mechanism that is inactivated by Bcl-xL and/or Mcl-1. It should be noted that apoptosis and senescence are believed to be generally exclusive and the manner in which cells select one of these two suppressive mechanisms is not clearly determined [15].

In this study, we further extend this observation, showing that the pharmacological inhibition of Akt prevented cell emergence *in vitro* and the growth of persistent cells *in vivo* in mice. Emergence was inhibited as a consequence of apoptosis induction, Noxa upregulation and binding to Mcl-1. Most significantly, this was observed in parallel with senescence inhibition and a reduced induction of p21waf1, the main mediator of CIS. Using either RNA interference or p21waf1-deficient cells, we also observed that the presence of an intact p21-senescence pathway allows cell emergence and that its downregulation improved the efficacy of the treatment. The same improvement was observed following p53 inactivation.

As we previously discussed [18], we need to consider the possibility that a subpopulation of resistant cells already present before the treatment could induce cell emergence. However, we consider this unlikely since the emergent cells have the same sn38 sensitivity as compared to parental cells [18]. In addition, they grow in low adhesion conditions whereas this is not the case of LS174T cells in our experimental conditions [18]. Note also that we obtained the same number of emergent cells if our experiments were performed in 10% or 3% serum (data not shown). Thus quiescent cells are probably not involved in treatment escape. Although this remains to be proved, our hypothesis is that sn38 induces a phenotypic switch that allows the emergence of a specific subpopulation. If true, this might also explain why the Akt inhibitors are still efficient after 4 days of treatment despite the fact that Akt phosphorylation returned to basal levels (Figure 1D). This phenotypic switch is expected to occur in a very low percentage of cells. If rare cells rely on Akt signaling after 2-3 days of treatment to regrow and induce emergence, it is certainly difficult to detect its activation by global western blot, in a small subpopulation. Therefore, it will be important to identify specific cell surface markers of PLD and PLS cells. This will determine if these cells are present before the treatment but it will also allow their tracking during the response. Using classical markers such as lgr5, CD133, CD44 or ALDH activity, we have described that persistence was not associated with an increase in cancer initiating cells [18]. Interestingly, it has been reported that Akt is activated in a specific subset of cancer stem cells that express CD133, CD44 and CD24 [55]. These cells are resistant to radiation-induced cell death. Further studies are needed to determine if these CD133^+^CD44^+^CD24^+^ cells are also involved in the resistance to sn38 and if they could define the PLD subpopulation.

In light of these observations and at least in response to irinotecan, it is tempting to conclude that apoptosis is a superior suppressive mechanism as compared to CIS. As stated in the introduction, it is not particularly clear whether these responses should be considered to be equal suppressive pathways [15]. Senescent cells produce soluble factors involved in cell migration, angiogenesis and EMT [12-14], and CIS can induce the failure of chemotherapy *in vivo* [16]. In line with this last study, our results suggest that inducing apoptosis increased treatment efficacy in comparison to CIS. Simply, one can consider that obtaining a dying tumor cell is a better treatment achievement than inducing a complete loss of replicative potential. If correct, this might also imply that p53-deficient tumors have a better response to irinotecan-based treatment, as recently proposed for breast tumors treated with doxorubicin [16]. As stated above, in the absence of this tumor suppressor, cancer cells progress more efficiently to mitotic catastrophy and apoptosis. Since multiple studies have found contradictory results on this subject [16], further studies will be necessary to define the role of the p53-senescence pathway in irinotecan-refractory colorectal cancers. We believe that a correct interpretation will probably be more complex. If senescent cells are effectively removed by immune cells *in vivo,* this was neither tested in our *in vitro* conditions nor using immuno-deficient mice. Thus a complete evaluation of senescence efficacy as compared to apoptosis relies on more complex experimental approaches. A more precise study of senescence as a tumor suppressive mechanism implies future experiments that incorporate immune anti-tumoral responses to study sn38 responses.

In addition to its phosphorylation in the early stage of CIS, we have observed that Akt is more active in the subpopulation of dividing PLD cells as compared to the senescent PLS cells (Figure 3C). Although this remains to be proved, this suggests that the benefit of Akt inhibition will depend on the heterogeneity generated within the residual tumor by irinotecan. Subpopulations emerging from chemotherapy that express less active Akt could be spared by this targeted therapy and remain viable. Thus, *in vivo* testing must be carried out on established tumors to determine whether this combined treatment elicits a complete eradication of cancer cells. In this case, sequential treatments should be used to allow -and then target- the outgrowth of emergent cells from the primary tumor. Note however that further experiments are also necessary to clarify the interactions between the PLS and PLD subpopulations. We speculate that a cooperation between these two subpopulations is necessary to sustain a survival niche. We believe that targeting Akt or a survival pathway within one subpopulation will affect the other.

**Figure 10:**
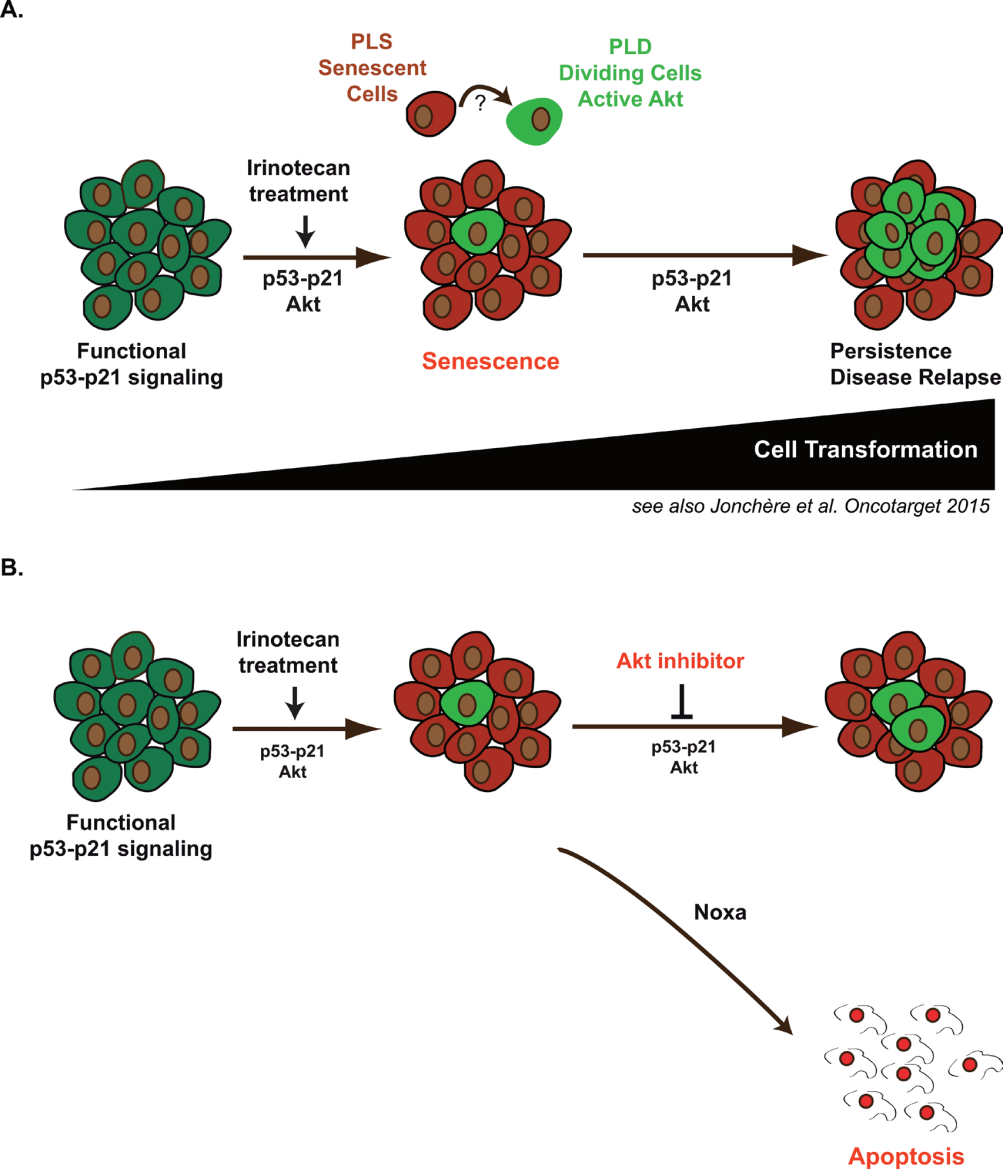
Emergence in response to sn38 is associated with increased Akt activation: apoptosis induction should reduce irinotecan and senescence failure in colorectal cancer In response to sn38, a small fraction of cells escapes the senescence suppressive arrest and emerges as an heterogeneous and more transformed population (see also reference 18). Akt is activated during the early step of this response and in the emergent dividing cells. Its presence favors cell emergence and its inhibition consequently improves sn38 efficacy by reducing the senescence suppressive arrest and activating apoptosis instead. We speculate that these emergent cells are responsible of treatment failure and disease relapse. Therefore, sequential Akt targeting should be considered in the future to improve the treatment of irinotecan-refractory colorectal cancers through the induction of apoptosis.

These results also illustrate the utility of combining a pro-apoptotic therapy with a first line treatment known to induce senescence. It has been recently demonstrated that these two mechanisms can cooperate *in vivo* to prevent the growth of Myc-driven tumors [56]. We therefore propose that Akt inhibitors might be useful to prevent cell emergence in response to irinotecan-based treatments (Figure 10). Although this remains to be demonstrated *in vivo*, sequential chemotherapy might be used to first induce senescence and secondly target the specific subpopulations that use the Akt rescue pathway. Inhibitors of the Akt and PI3K pathways have been tested in early stage clinical trials and it seems that their clinical efficacy is generally low when used as monotherapies [57]. Since Akt inhibition activates survival feedback pathways [27], combination therapies with lapatinib, trastuzumab or anti-oestrogen are undergoing clinical evaluation. Provided that the combination is not toxic, we therefore propose to test the hypothesis that Akt inhibitors reduce the occurrence of irinotecan-refractory colorectal carcinomas when used in sequential therapies.

## MATERIALS AND METHODS

### Cell lines and treatment

All cells were obtained from the American Type Culture Collection (except HCT116 p21−/− cells obtained from Dr B. Vogelstein). Cells were maintained in antibiotic-free RPMI 1640 medium (Lonza), supplemented with 10% fetal bovine serum and were routinely tested to rule out mycoplasma contamination. Cell treatments were performed in 3% FBS except for the detection of Akt activation (10% FBS to improve the Akt Ser473 blot, Figure 1). To induce persistent cell generation, LS174T cells were treated for 4 days with sn38 (5 ng/ml or 12.7 nM; Tocris Bioscience 2684) in 3% FBS, washed with PBS and then restimulated with fresh 10% FBS for 7 days. Note that generating persistent HCT116 took more time since cells do not regain their proliferative potential easily. In this case, cells were restimulated twice with fresh 10% FBS; after 4 days of sn38 treatment and after 7 days of emergence. Clones were generally counted after 2 to 3 weeks. Note also that emergence did not rely on the serum concentration, the same number of clones and the same p21waf1 expression can be observed in 10% or 3% FBS.

### Akt inhibitors

GSK690693 was obtained from GlaxoSmithKline and dissolved in DMSO at a concentration of 10 mM before use. For tumor xenograft studies, GSK690693 was formulated in 10% DMSO and used at 10 mg/kg. The Akt 1/2 kinase inhibitor was obtained from Sigma (A6730).

### SN38 concentrations and assays

Various assays were used in this study, either short time (flow cytometry, MTT, protein expression/interaction) or longer time such as clonogenic assay to visualize all treatment responses (mitotic catastrophy, senescence, long terme proliferation recovery or quiescence). IC50 concentrations were always determined by clonogenic assays, typical responses are presented Figure 1A (note that clonogenic test and IC50 value are thus not equivalent to a toxicity measurement). In short time assays (4 days) and in the emergence experiments, cells were plated at a higher concentration and are thus expected to be less sensitive to sn38 as compared to clonogenic tests. In these conditions, the topoisomerase inhibitor was always used at 5 ng/ml (12.7 nM), a higher concentration that is expected to target all cells (see Figure 1A). Note that this is also the concentration that is detected in the serum of the patients [58]. For short time assays, GSK690693 was used at 20μM, Akti 1/2 at 10μM.

### Clonogenic assay

Cells were seeded at 1500 (LS174T) cells or 500 (HCT116) cells into 6-well and incubated at 37°C in 5% CO_2_ atmosphere. One day later, the cells were treated with sn38 (0.15 to 1 ng/ml; 0.381 nM to 2.5 nM), GSK690693 (0.1 to 10 μM) and Akt ½ inhibitor (1 to 5 μM) for 7 to 10 days, washed twice with PBS, and stained with 0.4 % crystal violet. The colonies were then washed with water, visualized with a Bio-Rad Chemi Doc XRS Imaging device and counted using Quantity One Imaging software (Bio-Rad). The survival fraction was determined as the percentage of the treated colonies as compared to the number of colonies grown without drug.

### MTT assay

Cell viability was also measured by the MTT (3-4,5-dimethylthiazol 2,5-diphenyltetrazolium bromide) assay. Cells were seeded in 96-well clear-bottomed plates at a density of 2500 cells per well in 200 μL medium, incubated for 24 hrs, and treated with sn38 (5 ng/ml or 12.7 nM), GSK690693 (20 μM) and Akti ½ (10 μM) and their combination. Four replicates per concentration, per treatment were done. After 72 hrs, 40 μL of 5 mg/mL MTT solution was added to each well and incubated in a humidified 5% CO_2_ atmosphere at 37°C for 3 hrs. After incubation, the cells were pelleted and dried. Subsequently, 100 μL of DMSO (dimethylsulfoxyde) was added to each well, and the cells were incubated at room temperature for 2-3 hrs. Absorbance was measured by a microtiter plate reader at 562 nm.

### siRNA inactivation

To downregulate gene expression, cells were transfected with 50 nM CDKN1A ON-Target plus SMARTpool (Dharmacon), On-Target plus human TP53 (7157) si-RNA-SMART pool (Dharmacon), NOXA siRNA (Santa Cruz, sc-37305), Akt 1/2 si-RNA (Santa Cruz, sc-43609), prevalidated siRNA against Mcl-1 (Ambion AM51331 (ID :120642) and prevalidated control siRNA (5′-GCACUAACUACCGUGAUUATT-3′ and 5′-GAAAGAAGCACUCGUAUAATT-3′ from MWG) using DharmaFect-4(Dharmacon) according to the manufacturer's instructions.

### Tumor xenografts

Animal studies were conducted in strict accordance with the principles and procedures approved by the local Ethical Committee. Nude mice (BALB/C nu/nu) were inoculated subcutaneously with two million PLC cells at the age of 8 weeks. After 4 days, mice were treated or not with GSK690693 (10 mg/kg), 5 days per weeks. Control treatments were performed with DMSO (10%) as GSK690693 was dissolved in DMSO 10% at a concentration of 10 mg/kg. The tumor volume was monitored for 4 weeks. An electronic caliper was used to measure the length (L), width (W) and depth (D) of the tumors 5 times per week. Tumor volume was estimated by applying the following equation: 0.52 * L * W * D. Experiments were conducted using five mice per group.

### Western blotting

Following cell lysis at 4°C (25 mM HEPES, pH 7.9, 300 mM KCl, 0.2 mM EDTA, 10% glycerol, 1% NP40, 1 mM PMSF, 10 μg/ml aprotinin, 10 μg/ml leupeptin, 10 μg/ml pepstatin, 1 mM Na3VO4, 50 mM NaF), lysates were sonicated and then boiled for 10 min. Proteins were separated on a SDS polyacrylamide gel and transferred to a PVDF membrane. Following a 1 hr incubation in 5% BSA, Tris-buffered saline (TBS), and 0.1% Tween 20, the membranes were incubated overnight at 4°C with antibody.

The following antibodies were used: rabbit polyclonal anti phospho-Akti 1/2/3 (Thr308)-R (1:750; Santa Cruz sc-16646-R), rabbit monoclonal anti-phospho-Akt (Ser473) (193H12) (1:750, Cell signaling 4058), goat monoclonal anti-Akt1/2 (N-19) (1:1000; Santa Cruz-sc 1619), rabbit monoclonal anti-phospho-GSK3Δ Ser9 (1:750; Cell Signaling 9336), rabbit polyclonal anti-GSK3β (H-76) (1:1000, Santa Cruz sc-9166), rabbit anti- cleaved caspase 3 (Asp175)(1/1000; Cell signaling 9661), rabbit polyclonal anti-phospho-p53 (ser15)-R (1:1000, Santa Cruz sc11764-R), mouse monoclonal anti-p53 (Pab 1801) (1:500; Santa Cruz sc 98), rabbit monoclonal anti-p21 (1:1000; Cell Signaling 2947S), rabbit polyclonal anti-Mcl-1 (1:1000; Santa Cruz sc-819), mouse anti-Bcl-xL (1:1000, BD Bioscience 610747), mouse monoclonal anti-Noxa (1:1000; Enzo Life Science ALX-804-408-C100), mouse monoclonal anti-HSC70 (1:1000, Santa Cruz sc7298). Membranes were then washed twice with TBS with 0.1% Tween 20 and incubated for 1 h with peroxidase-conjugated secondary antibodies (Santa Cruz). Revelation was performed by chemiluminescence with a Bio-Rad Chemi Doc XRS imaging device (Bio-Rad).

### Co-immunoprecipitation assay

Cells were lysed in Lysis Buffer pH 7.4 (20mM Tris-HCl, 150mM NaCl, 1mM EDTA, 1% Triton X100, 1 mM PMSF, 10 μg/ml aprotinin, 10 μg/ml leupeptin, 10 μg/ml pepstatin, 1 mM Na3VO4, 50 mM NaF). Beads were precoated with 1μg of antibodies (rabbit polyclonal anti-Mcl-1 (Santa Cruz Sc-819) or rabbit polyclonal anti-Gal4 (Santa Cruz sc-729)) for 2h of rotation at 4°C. After two washes, 500 μg of cell lysate were added to pre-coated beads. Immunoprecipitation was carried out overnight under rotation at 4°C. After two washes with lysis buffer, beads were resuspended in 30 μL 2X sample buffer (10% SDS, 10% glycerol, 1 M Tris Base). Western blot was then performed as described above.

### Flow cytometry

*Dapi DNA Staining*—Cells were trypsinized, washed with PBS and 250 000 cells were fixed with 4% paraformaldehyde for 10min at 37°C, then washed and incubated with cold 70% methanol at 4°C for 30 min. After a cold passage, cells were washed and incubated with DAPI (5 μg/ml) in PBS-BSA 2%-Triton0.2% for one hour at room temperature. Cells were then analyzed by flow cytometry. *Ki67 staining*— Cells were trypsinized, washed with PBS and 500 000 cells were incubated with cold 70% methanol at 4°C for 30 min. After a cold passage, cells were washed twice with PBS-Tween 0.1% and PBS-BSA1%-Tween 0.1%. Then, cells were incubated 30 min at room temperature and in the dark with antibodies using the FITC Mouse anti-Human Ki-67 Set (BD Pharmingen, 556026). After washing with PBS-BSA 1%-Tween0.1%, DAPI DNA staining was performed as described above. *Cleaved Caspase 3 staining*— Cells were trypsinized, washed with PBS and 500 000 cells were fixed and permeabilized with BD Cytofix/cytoperm kit (BD Biosciences; 554714) according to the manufacturer's instructions. After washing with BD Perm/Wash 250 000 cells were incubated 30 min at room temperature and in the dark with 25ng of PE rabbit IgG anti-Active Caspase3 (BD; 550821) or 25ng of PE rabbit IgG (Cell signaling; 5742S) as isotype control. Cells were then washed with BD Perm/Wash and incubated with DAPI (5μg/ml) in PBS-BSA 2%-Triton0.2% for one hour at room temperature. Cells were then analyzed by flow cytometry.

### β-Galactosidase (β-Gal) staining

Cells were fixed for 15 min (room temperature) in 2% formaldehyde. After fixation, cells were washed with PBS and incubated at 37°C (without CO2) with freshly-made senescence associated β-Gal (SA-β-Gal) staining solution: 0.3 mg/ml of 5-bromo4-chloro-3-indolyl β-D-galactoside (X-Gal, Fermentas), 40 mM citric acid (Sigma), 40 mM sodium phosphate (Sigma) (stock solution (400 mM citric acid, 400 mM sodium phosphate) must be at pH 6), 5 mM potassium ferricyanide (Sigma), 5 mM ferrocyanide, 150 mM NaCl (Sigma) and 1.5 mM MgCl2 (Sigma). Staining was evident after 16-20 hr. Senescent cells display a perinuclear precipitation of blue dye, which can be observed with standard light microscopy. SA-β-GAL-positive were quantified by counting stained and unstained cells and expressed as the percent of positive cells over the total counted.

### PML immunofluorescence

Cells were fixed using 2% formaldehyde in phosphate-buffered saline (PBS) for 15 minutes at room temperature and were then permeabilized by incubating for 30 minutes in ethanol 70% at −20°C. After washing 3 times in PBS-0.02%Tween and blocking in PBS-5% serum during 15 min, the cells were incubated for 4 hour at room temperature with an anti-PML antibody (mouse monoclonal IgG_1_, Santa Cruz : sc-966, (1/50)) diluted in the blocking buffer. After washing 3 times in PBS-0.02% tween and blocking in PBS-5% serum during 15 min, cells were incubated with the secondary antibody 1/200 diluted (Goat anti-Mouse IgG secondary antibody Alexa 488, Invitrogen-Molecular Probes : A-10680) for 1 hour at room temperature in the dark. Cells were then washed 3 times in PBS-0.02%Tween, and covered with Anti-Fading reagent with DAPI (Invitrogen Prolong R. Gold antifade reagent with DAPI P36935). Slides were then analyzed by confocal microscopy.

### Statistical analysis

All values were expressed as mean +/− standard deviation (SD). Differences were analyzed using non parametrics tests (Mann-Whitney and Wilcoxon tests).*p < 0.05, **p < 0.01, and ***p < 0.001. No stars on the figure means that the result was not significant.

## Abbreviations

PLC: Persistent LS174T Cells.
